# Exploring Novel Innovation Strategies to Close a Technology Gap in Neurosurgery: HORAO Crowdsourcing Campaign

**DOI:** 10.2196/42723

**Published:** 2023-04-28

**Authors:** Philippe Schucht, Andrea Maria Mathis, Michael Murek, Irena Zubak, Johannes Goldberg, Stephanie Falk, Andreas Raabe

**Affiliations:** 1 Department of Neurosurgery Inselspital, Bern University Hospital University of Bern Bern Switzerland

**Keywords:** collective intelligence, crowdsourcing, fiber tracts, ideation, Mueller polarimetry, neuroscience, neurosurgery, open innovation, polarization

## Abstract

**Background:**

Scientific research is typically performed by expert individuals or groups who investigate potential solutions in a sequential manner. Given the current worldwide exponential increase in technical innovations, potential solutions for any new problem might already exist, even though they were developed to solve a different problem. Therefore, in crowdsourcing ideation, a research question is explained to a much larger group of individuals beyond the specialist community to obtain a multitude of diverse, outside-the-box solutions. These are then assessed in parallel by a group of experts for their capacity to solve the new problem. The 2 key problems in brain tumor surgery are the difficulty of discerning the exact border between a tumor and the surrounding brain, and the difficulty of identifying the function of a specific area of the brain. Both problems could be solved by a method that visualizes the highly organized fiber tracts within the brain; the absence of fibers would reveal the tumor, whereas the spatial orientation of the tracts would reveal the area’s function. To raise awareness about our challenge of developing a means of intraoperative, real-time, noninvasive identification of fiber tracts and tumor borders to improve neurosurgical oncology, we turned to the crowd with a crowdsourcing ideation challenge.

**Objective:**

Our objective was to evaluate the feasibility of a crowdsourcing ideation campaign for finding novel solutions to challenges in neuroscience. The purpose of this paper is to introduce our chosen crowdsourcing method and discuss it in the context of the current literature.

**Methods:**

We ran a prize-based crowdsourcing ideation competition called HORAO on the commercial platform HeroX. Prize money previously collected through a crowdfunding campaign was offered as an incentive. Using a multistage approach, an expert jury first selected promising technical solutions based on broad, predefined criteria, coached the respective solvers in the second stage, and finally selected the winners in a conference setting. We performed a postchallenge web-based survey among the solvers crowd to find out about their backgrounds and demographics.

**Results:**

Our web-based campaign reached more than 20,000 people (views). We received 45 proposals from 32 individuals and 7 teams, working in 26 countries on 4 continents. The postchallenge survey revealed that most of the submissions came from single solvers or teams working in engineering or the natural sciences, with additional submissions from other nonmedical fields. We engaged in further exchanges with 3 out of the 5 finalists and finally initiated a successful scientific collaboration with the winner of the challenge.

**Conclusions:**

This open innovation competition is the first of its kind in medical technology research. A prize-based crowdsourcing ideation campaign is a promising strategy for raising awareness about a specific problem, finding innovative solutions, and establishing new scientific collaborations beyond strictly disciplinary domains.

## Introduction

Gliomas are the most common type of primary brain tumors [1,2]. Surgical resection plays a central role in their management, and there is increasing evidence that the extent of tumor resection correlates well with overall and progression-free survival in patients with both high- and low-grade gliomas [3-5]. In recent decades, new techniques have become available to allow for more radical and safer brain tumor surgery. Intraoperative magnetic resonance imaging, ultrasound, and fluorescence guidance attempt to visualize tumor tissue [6-8]. Intraoperative monitoring helps to identify brain areas involved in motor and speech function [9-11]. However, each of these technologies has its drawbacks. Low-grade glioma and infiltration zones of high-grade glioma remain difficult to discern. Most neurological functions cannot be investigated by intraoperative monitoring, and for speech mapping, the patient is required to be awake during surgery. None of these techniques provides real-time feedback about fiber tract location or tissue delineation [12,13]. The ability to see white matter tracts live during surgery would help to differentiate white matter from tumor tissue based on the presence of fibers. In addition, being able to see the fibers would allow the neurosurgeon to identify and spare specific, crucial fiber tracts such as the arcuate fasciculus, the corticospinal tract, and the optic radiation due to their spatial orientation and to orientate himself or herself based on the direction of the fibers that can be seen. Such a technology would need to be noninvasive, nontoxic, and able to provide information about fibers and their spatial orientation in real time.

Innovative research, by means of high-risk projects, in medicine is often subject to tight constraints placed on scientists, such as funding difficulties and a culture of private endeavor rather than reaching out to others [14]. During the 20th century, innovations from the fields of chemistry, physiology, and physics revolutionized medicine. Technological advancements and interdisciplinary research have become indispensable in the quest for improvement in modern medicine [15]. Investigator isolation, by contrast, impedes collaboration and thus hampers progress [16].

The development of the Web 2.0 technologies around the turn of the millennium enabled internet users to act as both consumers and contributors of content and to connect to each other independent of location [17]. This opened up completely new ways of collaborating and paved the way toward exploiting the wisdom of the crowd for innovation and research. The concept designated “crowdsourcing,” a portmanteau composed of “crowd” and “outsourcing,” coined by Jeff Howe [18] in 2006, relies on accomplishing a task by opening up its execution to the broad public crowd [19,20]. The advantages of crowd participation have been exploited for centuries, starting in 1714, when the British Government offered £20,000 (US $24,642.40) to anyone who could find a way to calculate the longitudinal position of a ship. The problem was solved in 1730 by John Harrison, a carpenter and clockmaker, who presented the first sea clock (chronometer) [21]. And 300 years later, billions of people are connected via the internet, enlarging the crowd for crowdsourcing enormously. This not only opens up access to much more “crowd intelligence,” but also enables networks and collaborations across geographic boundaries and across a plethora of research teams from a vast variety of scientific fields. The model takes advantage of the wisdom of the crowd and counteracts the silo mentality and secrecy traditionally associated with classical research and development [22].

In health and medical research, crowdsourcing has evolved over the past few decades. Strategies that include the public or a specialist community are broadly applied to recruit patients, collect data, generate intellectual output, conduct evaluations, gather new ideas, or solve specific problems together [23-38]. Crowdsourcing in the form of open innovation challenges was reported by the National Aeronautics and Space Administration (NASA) [39] and the Obama administration [40]. Independent of the type of crowdsourcing applied, the concept has been shown to save time and money, as well as spur innovation [41-43].

Motivated by these findings and convinced that the solution to our problem already existed beyond the community of medical professionals, we turned to the public to catalyze interdisciplinary research and development. In the search for an innovative solution to overcome a longstanding technical dilemma, we launched “*HORAO—The It Doesn’t Take a Brain Surgeon*” challenge. Despite considerable evidence supporting the effectiveness of open innovation as an alternative method in health and medical research, to our knowledge, ours was the first open innovation challenge of its kind.

## Methods

### Overview

We designed a multiphase, prize-based, open innovation competition in collaboration with the commercial platform provider HeroX. The challenge page on HeroX [44] served as a content hub throughout the challenge. It featured an explanatory video and a text-based description of the technology gap and its background. On HeroX, we published the judging scorecard and all formal and legal requirements. Moreover, the challenge hub included a chat room for discussions and questions for the sponsor team. The whole of the financial funds previously collected in a crowdfunding campaign were used for the crowdsourcing campaign [45]. First, expenses for using the HeroX platform, production of informative media content (eg, the video), and organization of the HORAO conference, as well as the travel costs of the participants and the jury, were paid. The remaining US $50,000 served as the monetary incentive for putting forward existing solutions. We decided to divide this prize money among several finalists to increase the likelihood of any innovator winning a prize and further motivate innovators to participate. The final share was set at US $35,000 for the winner, US $12,000 for the runner-up, and US $1000 each for the third to fifth places.

### Prelaunch

**Figure 1:**
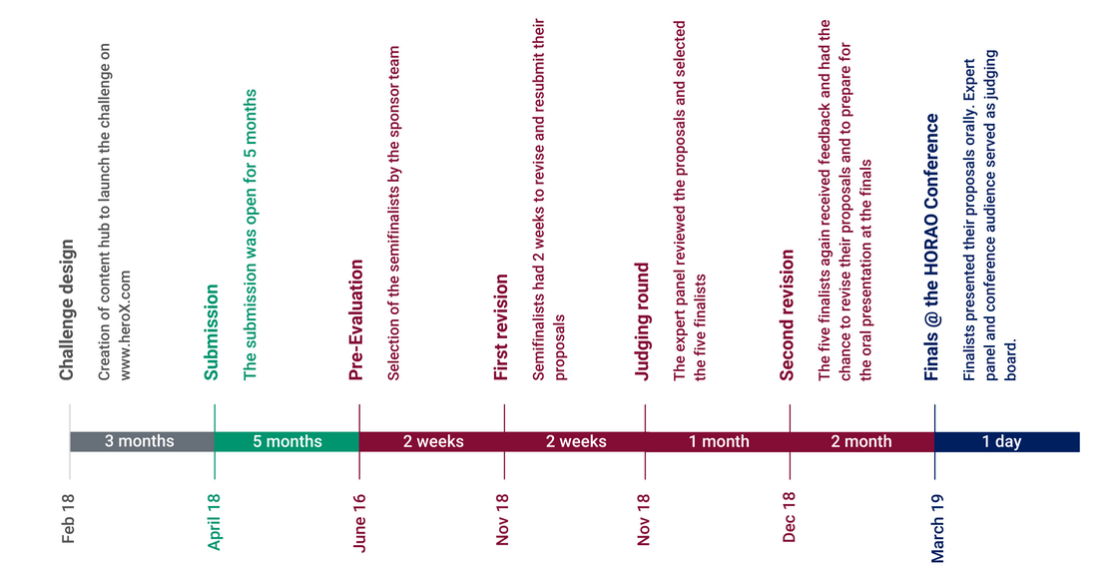
Challenge timeline: the challenge was designed in a stepwise approach developed in the prechallenge phase over 3 months (challenge design). The phase for submission was the longest along the challenge timeline. After submission was closed, an evaluation-feedback-revision loop started. This had 2 phases (preevaluation and judging round).

After 3 months of content creation (video and text), on April 23, 2018, the challenge went on the internet with a prelaunch in the categories of engineering, health care, and technology. The aim of the prelaunch phase was to raise awareness about the ensuing competition and give solvers a first opportunity to evaluate the challenge. HeroX’s service included advertisements on Facebook (2 weeks) and Twitter (1 week) as channels for recruiting potential participants and a one-on-one outreach campaign (targeted outreach: 2108 emails). The target audience for the one-on-one outreach included individuals, companies, and organizations involved in medical imaging, medical technology, radiology, surgical technology, clinical engineering, neurological societies, imaging science, health science, and microscopy. Finally, HeroX published the HORAO challenge in its newsletter as part of the media service. Figure 1 provides an overview of the challenge timeline.

### Submission Phase

The submission phase started on June 12, 2018, and ended on November 16, 2018 (after 22 weeks and 4 days). Proposals had to be presented using the official submission form and to address the questions asked thoughtfully. The form had space for a technical report with the option to embed a link to a video or website. The complete proposal had to be uploaded as a PDF to HeroX. Submissions needed to comply with all the terms of the challenge defined in the challenge-specific agreement, which specified, for example, that competitors retain all intellectual property rights to their technology. The challenge was open to all adult individuals or teams, requiring no specific qualifications. We considered, for further evaluation, only submissions satisfying the criteria of the judging scorecard (Table 1). We made the judging scorecard and the challenge guidelines publicly accessible from the very beginning. The judging scorecard narrowed down and specified the scope of the possible solution. The challenge guidelines and the challenge-specific agreement are reproduced in Multimedia Appendices 1 and 2.

After termination of the submission phase, the challenge team launched an individual project website serving as a new content hub independent of HeroX. The sponsor team also published regular updates about the challenge on the hospital and departmental websites and via social media channels (Facebook, Twitter, and Instagram).

**Table 1:** Judging scorecard published together with the challenge guidelines. The predefinition of the judging scorecard narrowed down and specified the scope of the possible solution with the aim of reducing submissions of unsuitable solutions.

Section	Description	Overall weight
Detection of cerebral tissue	The solution discerns brain from tumor tissue	20
Detection of fiber tracts	The solution detects brain tissue in such a way that the spatial orientation of fiber tracts can be seen	20
Real-time detection	Time used for visualization must be short (minutes) in order not to disrupt the flow of surgery	20
Size of solution	The size of the solution must be such that it fits well into the operating theater (not larger than 2 cubic meters)	10
Noninvasiveness	The solution must not harm or remove the investigated tissue	20
Repetitiveness	The solution must be able to be used repetitively at short interval (minutes)	10

### Evaluation Phase

The evaluation phase consisted of the following 3 consecutive rounds: the preround, the judging round, and the finals. Each round had its own panel of judges.

#### Preround Evaluation

In the preround evaluation, the sponsor team, consisting of 4 neurosurgeons, formed the panel of judges. The sponsor team performed a first evaluation of all proposals based on the judging scorecard criteria. The aim of the preround was to facilitate the work of the expert panel by rejecting proposals that did not meet the criteria and limiting the number of proposals to 10-15. The votes of at least 2 out of the 4 members of the sponsor team were required for the proposal to be chosen for the next round. The sponsor team gave feedback to those selected for the judging round about their submissions. Participants then had to resubmit the revised proposals within 2 weeks.

#### Judging Round Evaluation

An expert panel, consisting of 2 research and development directors from medical technology companies, 2 neurosurgeons, and 3 senior biomedical scientists, assessed the proposals in the judging round. Each member of the expert panel rated the proposals that passed the pre-evaluation by awarding a certain number of points (0 to max weight; Table 1) for each of the criteria. The 5 submissions with the highest score entered the finals. Again, the sponsor team gave feedback about the selected proposals based on the jury’s assessment, and the finalists had the option to revise their submission prior to the finals.

#### Finals

The finals took place at the HORAO conference on March 15, 2019, which was open to the public. For the finals, the expert panel from the judging round and the audience formed the judging board. Each finalist presented his or her proposal orally and then answered questions from the audience and the expert panel. The final score consisted of the points awarded by the expert panel in the judging round (50%), the score given for the presentation at the final conference by the expert panel (25%), and the score given for the presentation by the general audience (25%).

### Postchallenge Analysis

We refrained from asking for background information about the solvers themselves to avoid discouraging those with a lower level of academic attainment from submitting solutions and to avoid selection bias during the judging round. Using a web-based survey (SurveyMonkey [46]), sent to the individuals and teams, we obtained this information after the finals. We asked team leaders to forward the survey to their team members to capture information about as many of them as possible. The questions in the survey covered place of residence, type of employment and place of work, academic degrees obtained and field of education, number of prior challenges joined, and how the solver found out about the HORAO challenge. We used descriptive statistics to evaluate the diversity of the solvers crowd. In addition, HeroX performed an analysis of the reach of advertising on social media channels (Facebook and Twitter) for challenge visibility as part of their service.

### Ethical Considerations

We did not use any health-related data for this work. Participants shared the information about educational background and demographics voluntarily. We treated all personal data disclosed with the utmost care. The project does not fall under the jurisdiction of the local ethics committee, so we did not need to obtain their approval.

## Results

### General Crowd and Solvers

#### Preround Evaluation and Solvers Crowd

The challenge on HeroX attracted 20,680 views, and 274 individuals and 17 teams actively followed the challenge hub. The first and second advertisements published by HeroX on Facebook were displayed to 10,751 and 4466 users, respectively, and the advertisement on Twitter to 23,718 users. Eighty-one and 46 users actively clicked the link in the 2 advertisements published on Facebook, and on Twitter, 109 users actively clicked the link. Overall, 2108 individual emails were sent in the targeted outreach performed by HeroX.

**Figure 2:**
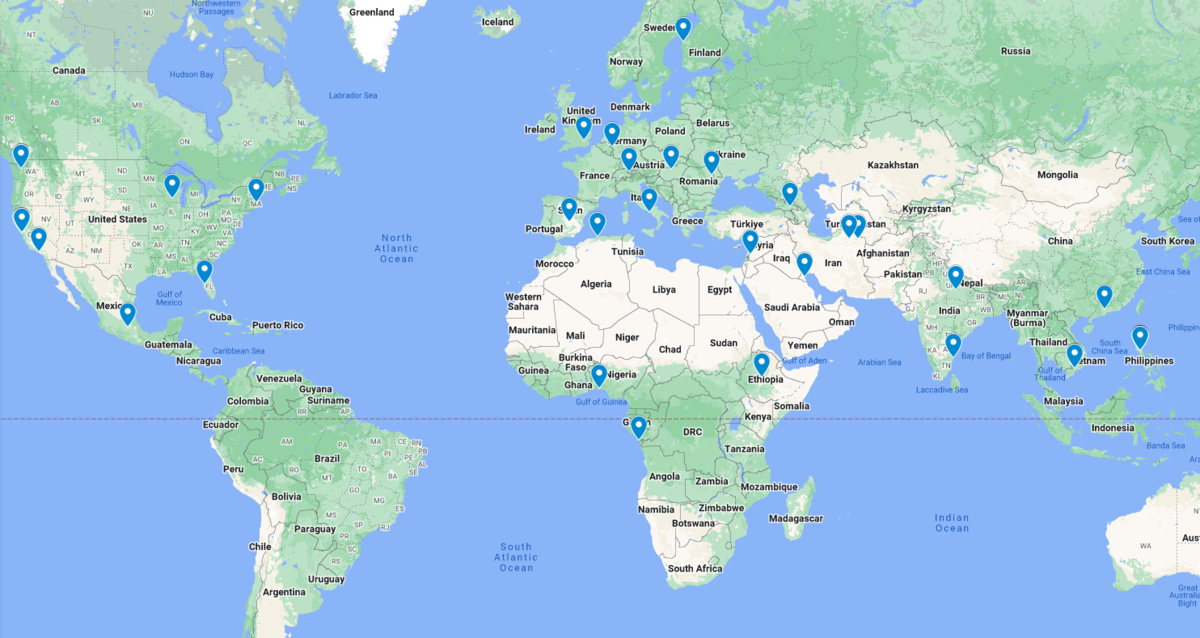
The majority of the 45 submissions were from North America, Europe, or Asia. One solver from Manila, Philippines, submitted 4 proposals. Two submissions were from Seattle, USA, and 2 from Ho Chi Minh City, Vietnam.

A total of 45 proposals were submitted by 7 teams and 32 individuals. All members of the 7 teams and the 32 individuals formed the *solvers crowd* of the challenge. The background survey was sent to 39 individuals, and feedback was obtained from 23 (58.9%). If we received no response, we searched their profile on HeroX or LinkedIn for any information. Finally, we collected background information about 39 solvers. Four people were involved in more than one submission. For further analysis of the crowd, we treated them like individual solvers of every submission, resulting in a crowd of 45 solvers. A total of 4 women and 41 men aged between 16 and 75 years from 26 countries on 4 continents formed the solvers crowd (Figure 2). Most of the solvers were from Asia, North America, or Europe. A total of 37 solvers reported having a university degree (18 bachelor’s, 9 master’s, 9 PhDs, and 1 professor), 1 reported no degree and 7 solvers did not provide any information about an obtained degree. Solvers reported an educational background in the field of engineering (13/45, 28.9%), natural sciences (12/45, 26.7%), technology (4/45, 8.9%), or a nonrelated field like finance, architecture, or other (16/45, 35.5%). Seven solvers did not provide any information about their educational background. Most of the solvers were employed either at a university (10/45, 22.2%) or in industry (14/45, 31.1%); others reported to be self-employed (9/45, 20%). One solver was a student, three reported being freelancers or not employed, and one reported being retired. Three solvers reported being employed but not where, and four solvers did not provide any information about their employment. The solvers were working in the following areas: natural sciences (10/45, 22.2%), technology (9/45, 20%), engineering (9/45, 20%), aerospace (2/45, 4.4%), innovation ideation (2/45, 4.4%), or a nonrelated area like architecture, finance, or other (8/45, 17.8%). Five solvers did not report in which area they were working.

#### Judging Round Evaluation and Semifinalists

The sponsor team selected 13 submissions from 9 individuals and 3 teams for the judging round, forming the *semifinalists’ crowd*. The crowd composition with regard to current employment was comparable with that of the solvers crowd. The educational backgrounds of the semifinalists, with the exception of 1 member, were in the field of natural sciences or engineering. All of the semifinalists were familiar with innovation challenges prior to HORAO. The semifinalists originated from Europe, North America, and Asia.

#### Final Evaluation and Finalists

The expert panel selected the proposals of 1 woman and 4 men. All reported having an academic degree of at least bachelor’s level in the field of natural sciences or engineering. Of the 5 finalists, 3 reported that they worked in an academic research group in the field of mathematical oncology, bioengineering, or technology. One reported being self-employed, and 1 worked in industry, both in the fields of technology. The self-employed solver further reported having participated in about 100 challenges prior to HORAO, whereas the other 4 reported having participated in 1-4 prior challenges. The finalists came from Canada, the United States, the United Kingdom, Germany, and Spain. Table 2 provides an overview of the demographic and background characteristics of the 3 different crowds.

**Table 2:** Overview of the background and demographic characteristics of the different crowds.

		All (N=45), n	Semifinalists (n=13), n	Finalists (n=5), n
**Sex (unknown: n=0)**				
	Female	4	1	1
	Male	41	12	4
**Continent (unknown: n=0)**				
	Europe	10	4	3
	Asia	15	2	0
	North America	16	7	2
	South America	0	0	0
	Africa	4	0	0
**Field of education (unknown: n=7)**				
	Engineering	13	8	2
	Natural science	12	4	3
	Technology	4	0	0
	Architecture	3	0	0
	Finances	2	0	0
	Aerospace	1	0	0
	Other^a^	3	1	0
**Field of work (unknown: n=5)**				
	Engineering	9	5	0
	Natural science	10	3	3
	Technology	9	3	2
	Architecture	3	0	0
	Finances	1	0	0
	Aerospace	2	0	0
	Innovation ideation	2	1	0
	Other^a^	4	1	0

^a^“Other” includes international studies, communication, consultancy, and law.

### Outcome

The 5 finalists proposed solutions based on multispectral time-resolved fluorescence spectroscopy, polarization-sensitive optical coherence tractography, machine-learned interpretation of red-green-blue images, using polarized light based on Mueller polarimetry, and wide-field Mueller polarimetry based on Lu-Chipman decomposition. Of these, wide-field Mueller polarimetry based on Lu-Chipman decomposition received the highest score, both from the conference audience and the expert jury, and won our crowdsourcing ideation campaign. The solution uses a series of liquid crystals to polarize white light from a xenon light source and captures the polarization details of the tissue in reflection transfiguration. Following the conference, we initiated an in-depth scientific collaboration between the sponsor team and the winning research team. For their preliminary results, the collaborators were awarded an industry grant. Following a series of ex-vivo experiments on cadaveric animal brain tissue and on fresh human tumor tissue with a prototype of the Mueller polarization microscope [47,48], we created a new interdisciplinary research unit, which comprises neurosurgeons, optical physicists, neuropathologists, and experts on artificial intelligence. The group recently launched a multiyear, in-depth clinical project, which has been awarded a Swiss National Science Foundation *Sinergia* grant 205904. After 3 years of the HORAO conference, we have already published a series of promising preclinical results [49-53].

## Discussion

### Overview

With the crowdsourcing challenge HORAO, a technology-gap-type problem in neurosurgery was presented for the first time to the public, with the conviction that a solution already existed somewhere, albeit developed for a different use. The challenge proved successful, producing a handful of very innovative and promising proposals, leading to new scientific collaborations.

In recent years, simplified access for patients, participants, scientists, and biomedical staff through Web 2.0 has opened up a new world for collaborating on a variety of tasks. Consequently, various types of crowdsourcing have evolved in health care. A well-represented use case described in the recent literature concerns projects searching for new biomarkers, like the Anti-PD-1 Response Challenge, Prostate Cancer DREAM challenge, and the Multiple Myeloma DREAM challenge, to name but a few [24,35,36]. In those challenges, the initiators share data, usually from a large set of patients, on an open platform and mobilize groups of people with same interests around the world to analyze the data. Leveraging worldwide expertise and the power of the mass has speeded up the identification of novel biomarkers tremendously compared to the classical approaches used in research. Another, completely different approach, evaluated the Berlin Institute of Health, was the OPEN project [33]. In response to the slow progress being made with artificial pancreas systems for people with diabetes, the patient community has taken the problem into its own hands. Under the hashtag #*wearenotwaiting*, patients and their families are building their own systems and making the algorithms publicly available (do-it-yourself artificial pancreas systems, OpenAPS) [37]. The OPEN project examines what academia, industry, and individuals with diabetes can learn from each other by establishing empirical evidence of the impact of do-it-yourself artificial pancreas systems. The initiators of the OPEN project are convinced that such an interdisciplinary and collaborative approach will have a profound impact, not only on the patients but also on the health care system and on society in general. With the rise of machine learning in medicine, labeled data sets are in high demand. Hence, crowdsourcing for data labeling has become popular. A recent example is the NuCLS study [23]. This study used a crowdsourcing approach for nucleus classification, localization, and segmentation in hematoxylin- and eosin-stained slides of breast carcinomas using a preannotated data set elaborated in a previous crowdsourcing study [38]. The organizers specifically addressed medical students and graduates in pathology by searching interest groups on social media (Facebook and LinkedIn) and assigning the tasks depending on experience. The mixed crowd of experts and undergraduates produced the final NuCLS data set containing more than 220,000 annotations of cell nuclei. Although it was successful, the initiators of the project pointed out that the context-dependency of data set curation makes transfer of the approach to other problems difficult. The crowdsourcing approach described here focuses on the ideation process and therefore differs considerably from the abovementioned crowdsourcing applications.

**Figure 3:**
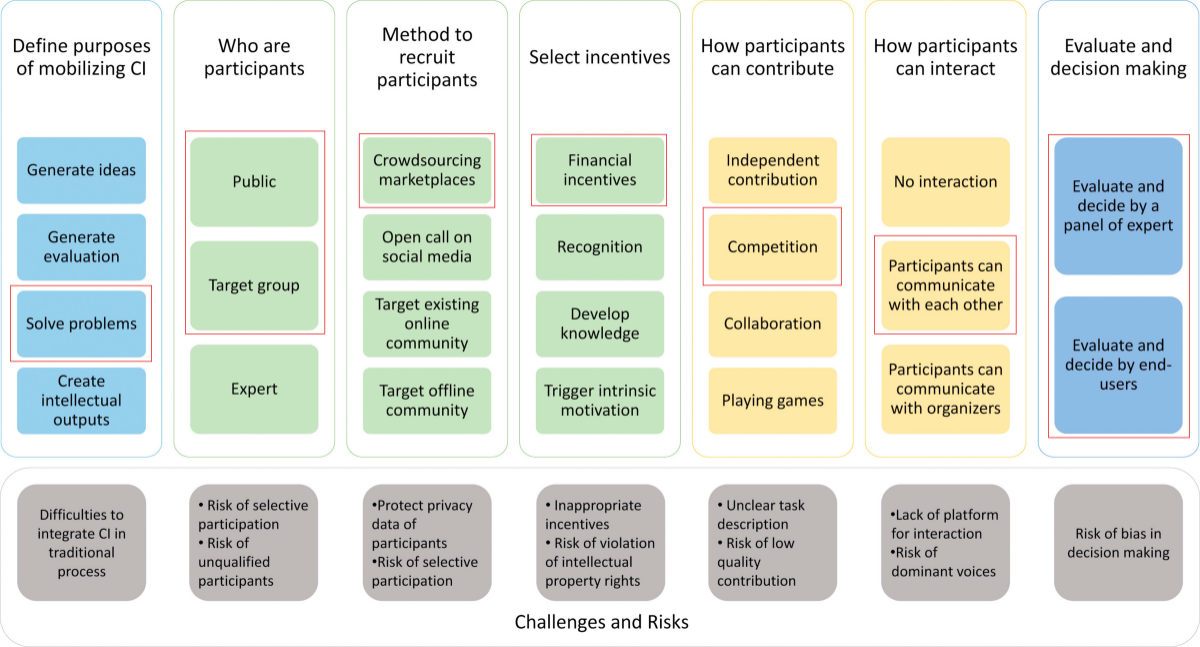
Framework of process of mobilizing collective intelligence (CI) (adapted from Nguyen et al 2019 [42]). The framework covers the major domains for planning and executing a crowdsourcing challenge. For each domain, the item which encounters the design used for HORAO is marked with a red square. Where two items are marked, HORAO used a combined approach of those two items.

The review by Nguyen et al [42] discusses the various methods of collective intelligence applied in clinical research and proposes a framework to implement them with respect to the domains shown in Figure 3. In comparison to other reviews that address crowdsourcing in medicine more generally [19,20,41,54], Nguyen et al [42] specifically addressed crowdsourcing that involved intellectual thinking on the part of the crowd and excluded other approaches like data collection or the performance of simple tasks (eg, classifying images or transcribing data). Information on ideation based on crowdsourcing is still scarce or underreported in the literature [42]. Only a pilot study by NASA [39] stands out. Since we were not able to identify other comparable projects, we discuss in detail the HORAO project in relation to the pilot projects of NASA, using the framework proposed by Nguyen et al [42].

### Purposes

HORAO was launched to overcome a technology gap in neuro-oncological surgery, specifically the inability to identify white matter tracts in real time during surgery. The rationale for turning to the public was that a solution already existed somewhere in another field of research, albeit having initially been developed to solve a different technological problem. The motivation to seek a solution, which many research groups have so far been unable to find, however, goes beyond simple ideation. The challenge organizers, all neurosurgeons themselves, face is the consequences of the lack of an appropriate technology almost daily, creating a strong personal desire to have a solution at hand. NASA faced a strategic challenge caused by a 45% reduction in its research and development budget in 2005. Formerly famous for its track record in research and innovation achieved by its own researchers, NASA decided to reach out to the crowd to solve space exploration problems, running a pilot program of challenges on InnoCentive [55] (NASA innovation pavilion). In NASA’s case, as in ours, the problem-owners identified a technology gap and believed that solutions for closing the gap would be accessible through open innovation.

### Participants, Recruitment, and Incentives

HORAO—like the NASA pilot challenges—was open to the public with no restrictions or specific requirements for solvers. In both projects, the monetary incentives were large enough to motivate solvers to apply known solutions without having to finance scientific investigations (average NASA: US $7500-$30,000; HORAO: CHF 1000-35,000 [US $1089.60-$38,136]). For recruitment and challenge execution, HORAO collaborated with HeroX, whereas NASA worked with InnoCentive. InnoCentive, launched in 2001, is the pioneer and longtime leader in its field. Its hallmark is the wide network of registered experts with various academic backgrounds (about 390,000 solvers, 60% with a master’s degree or higher) and its longstanding experience in crowdsourcing innovative research. HeroX was founded in 2013 with the intention of opening up access to the public, enabling them to participate in and contribute to innovation challenges (about 170,000 solvers). Both platform providers offer a range of services, from challenge conceptualization to pre-evaluation of submissions. The services offered by HeroX and InnoCentive are comparable. One of the major benefits for HORAO was the targeted outreach offered by HeroX, which sent announcement emails to potential participants specifically to raise their awareness of the challenge. Whereas InnoCentive charges a challenge fee of about CHF 75,000 (US $81,720), HeroX charges 18% of the prize money with the security of a 50% refund if no winning idea can be identified and of a 100% refund if no idea at all is submitted, making the platform attractive to first-time users.

The number of solvers attracted by the HORAO challenge was about half the number of followers recorded for the single NASA pilot challenges (HORAO: n=220; NASA: n=419). Based on the report of NASA’s pilot program by InnoCentive [56], an average challenge usually attracts about 300 followers. What is interesting, however, is that the final number of submissions was about the same (HORAO: n=45; NASA: n=11-108). HORAO solvers were from 26 different countries, while NASA pilot challenge participants were from 5-20 different countries for a single challenge and for all 7 pilot challenges, from 30 different countries. Most submissions came from the same continents—North America, Europe, and Asia. Although more than half (7/13, 53%) of the submissions that reached the judging round (semifinals) of the HORAO challenge came from North America, all 5 finalists originated from a different country on 2 continents (Europe and North America). The NASA pilot challenge reports diversity of solvers with regard to education and expertise on the level of all followers, whereas HORAO evaluated participants from the solvers to the finalists. Thus, the numbers are not directly comparable. Moreover, different categorizations of the fields of expertise were used, which makes direct comparison difficult. Solvers attracted by the HORAO challenge were educated or employed in the fields of engineering, natural sciences, or technology, with engineering as the leading discipline. Likewise, for the NASA challenges, engineering was also the major field of employment of the solvers, followed by computer and physical sciences. Of the NASA solvers, 30% (147/490) reported having expertise within the challenge’s discipline.

### Participants’ Contribution and Interaction

In these domains too, the crowdsourcing approach applied for HORAO matches the approach used for the NASA pilot challenges. Participants contributed by providing their ideas or solutions in a competitive manner, with repeated interactions with the challenge organizers and only minor interaction with each other. A limitation reported for the challenge design of the NASA pilot program was the lack of a user template for submitting a proposal. HORAO provided a predefined format (submission form) (Multimedia Appendix 3) with questions concerning the issues relevant for evaluation.

### Evaluation and Decision-making

Both the NASA pilot challenges and HORAO applied a stepwise evaluation system, with a first prescreening and consecutive evaluation round(s) conducted by an expert panel. In contrast to HORAO, in the NASA pilot challenges, the platform provider performed the prescreening. NASA itself reported this approach to be prone to inappropriate rating by the platform providers. The organizers of the NASA challenge resolved this issue by jointly defining clear rating criteria. The benefit of letting the platform providers do the pre-evaluation is that it lifts the burden of evaluating masses of low-quality proposals from the challenge owners, but it necessitates very accurately defined evaluation criteria. For HORAO, the judging scorecard was developed prior to the challenge, and the sponsor team performed a pre-evaluation, which prevented inappropriate rating and rejection of promising proposals.

In each case, pre-evaluation removed low-quality submissions and avoided the jury being overwhelmed in the consecutive evaluation round(s) by too many proposals, thus allowing them to focus on the promising solutions. For HORAO, an additional benefit of reducing the number of proposals passing the pre-evaluation stage was that it enabled an effective feedback system during the consecutive challenge phases.

### Difficulties

Most of the potential difficulties listed by Nguyen et al [42] (Figure 3) did not apply to HORAO (integration of open innovation in traditional processes and protection of data privacy) or were avoided by the choice of the challenge design (risk of unqualified solvers or low-quality proposals, inappropriate incentives, lack of platform, unclear task description, and risk of dominant voices). Nevertheless, selective participation and bias in decision-making are important risks to consider. Although open to the public, the targeted outreach by HeroX probably led to a partial selection of the participants. We do not consider this a major disadvantage since it raised awareness among the crowd addressed without excluding anyone from participating. In a future crowdsourcing ideation campaign, we would again focus the targeted outreach on the scientific crowd, possibly by advertising the campaign at specific conferences. The provision of predefined criteria was intended to avoid bias in decision-making or evaluation. Nevertheless, some criteria were more objectively assessable than others, creating a possible source of bias. Overall, the sponsor team greatly benefited from working with a preexisting commercial crowdsourcing company, especially as it was the team’s first such campaign.

### Limitations

Our strategy of crowdsourcing ideation relied on prize money as an incentive, to which the usual, especially governmental, funding agencies are unlikely to contribute. Collecting the prize money beforehand in a crowdfunding campaign, as done for the HORAO project, requires time and effort.

Running the crowdsourcing campaign also involved time-consuming tasks, such as producing explanatory videos, press releases, and daily responses to solvers’ questions. The performance of these tasks by the challenge owners themselves, who were laypersons in the case of HORAO, increased the time and effort expended because they first had to acquire the essential skills. Alternatively—and as done for the video in our project—some of these tasks may be outsourced, in which case they incur additional costs. The pre-evaluation performed by the sponsor team was another time-consuming task, limiting the future usability of the approach applied.

### Conclusion

The HORAO campaign was the first of its kind to crowdsource for new ideas on contemporary problems in neurosurgery. The campaign was successful in raising awareness of a longstanding neurosurgical problem; the lack of a means of intraoperative real-time visualization of fibers to delineate tumor tissue from surrounding healthy tissue. It allowed us to gain access to a multitude of outside-the-box potential solutions, and the team of experts was able to rapidly assess them in parallel for their capacity to solve our problem. Ultimately, the crowdsourcing campaign led to the creation of a very successful interdisciplinary research unit, which is now funded by traditional governmental funds.

## Appendix

Multimedia Appendix 1 Challenge guidelines.

Multimedia Appendix 2 Challenge legal agreement.

Multimedia Appendix 3 Submission form (empty).

## Abbreviations

NASA: National Aeronautics and Space Administration

## Data Availability

The data sets generated and analyzed during this study that are not displayed in this manuscript or its appendices are available from the corresponding author upon reasonable request.

## References

[1] Ostrom QT, Gittleman H, Stetson L, Virk SM, Barnholtz-Sloan JS (2015). Epidemiology of gliomas. Cancer Treat Res.

[2] Louis DN, Perry A, Reifenberger G, von Deimling A, Figarella-Branger D, Cavenee WK, Ohgaki H, Wiestler OD, Kleihues P, Ellison DW (2016). The 2016 World Health Organization classification of tumors of the central nervous system: a summary. Acta Neuropathol.

[3] Scerrati M, Roselli R, Iacoangeli M, Pompucci A, Rossi GF (1996). Prognostic factors in low grade (WHO grade II) gliomas of the cerebral hemispheres: the role of surgery. J Neurol Neurosurg Psychiatry.

[4] Hervey-Jumper SL, Berger MS (2016). Maximizing safe resection of low- and high-grade glioma. J Neurooncol.

[5] Lacroix M, Abi-Said D, Fourney DR, Gokaslan ZL, Shi W, DeMonte F, Lang FF, McCutcheon IE, Hassenbusch SJ, Holland E, Hess K, Michael C, Miller D, Sawaya R (2001). A multivariate analysis of 416 patients with glioblastoma multiforme: prognosis, extent of resection, and survival. J Neurosurg.

[6] Schucht P, Beck J, Abu-Isa J, Andereggen L, Murek M, Seidel K, Stieglitz L, Raabe A (2012). Gross total resection rates in contemporary glioblastoma surgery: results of an institutional protocol combining 5-aminolevulinic acid intraoperative fluorescence imaging and brain mapping. Neurosurgery.

[7] Senft C, Bink A, Franz K, Vatter H, Gasser T, Seifert V (2011). Intraoperative MRI guidance and extent of resection in glioma surgery: a randomised, controlled trial. Lancet Oncol.

[8] Stummer W, Reulen HJ, Meinel T, Pichlmeier U, Schumacher W, Tonn JC, Rohde V, Oppel F, Turowski B, Woiciechowsky C, Franz K, Pietsch T, ALA-Glioma Study Group (2008). Extent of resection and survival in glioblastoma multiforme: identification of and adjustment for bias. Neurosurgery.

[9] De Witt Hamer PC, Robles SG, Zwinderman AH, Duffau H, Berger MS (2012). Impact of intraoperative stimulation brain mapping on glioma surgery outcome: a meta-analysis. J Clin Oncol.

[10] Schucht P, Seidel K, Jilch A, Beck J, Raabe A (2017). A review of monopolar motor mapping and a comprehensive guide to continuous dynamic motor mapping for resection of motor eloquent brain tumors. Neurochirurgie.

[11] Raabe A, Beck J, Schucht P, Seidel K (2014). Continuous dynamic mapping of the corticospinal tract during surgery of motor eloquent brain tumors: evaluation of a new method. J Neurosurg.

[12] D'Amico RS, Englander ZK, Canoll P, Bruce JN (2017). Extent of resection in glioma-a review of the cutting edge. World Neurosurg.

[13] Almekkawi AK, El Ahmadieh TY, Wu EM, Abunimer AM, Abi-Aad KR, Aoun SG, Plitt AR, El Tecle NE, Patel T, Stummer W, Bendok BR (2020). The use of 5-aminolevulinic acid in low-grade glioma resection: a systematic review. Oper Neurosurg (Hagerstown).

[14] Johnston SC, Hauser SL (2008). Transformative research. Ann Neurol.

[15] Bronzino J, Enderle JD, Bronzino JD, Blanchard SM (2005). 1 - Biomedical engineering: a historical perspective. Introduction to Biomedical Engineering. 2nd ed.

[16] Johnston SC, Hauser SL (2008). Investigator balkanization. Ann Neurol.

[17] Hudson-Smith A, Batty M, Crooks A, Milton R (2009). Mapping for the masses: accessing Web 2.0 through crowdsourcing. Soc Sci Comput Rev.

[18] Howe J (2006). The rise of crowdsourcing. Wired.

[19] Ranard BL, Ha YP, Meisel ZF, Asch DA, Hill SS, Becker LB, Seymour AK, Merchant RM (2014). Crowdsourcing--harnessing the masses to advance health and medicine, a systematic review. J Gen Intern Med.

[20] Tucker JD, Day S, Tang W, Bayus B (2019). Crowdsourcing in medical research: concepts and applications. PeerJ.

[21] Sobel D (1995). Longitude: The True Story of a Lone Genius Who Solved the Greatest Scientific Problem of His Time.

[22] Chesbrough H (2006). Open Innovation: The Imperative for Creating and Profiting From Technology.

[23] Amgad M, Atteya LA, Hussein H, Mohammed KH, Hafiz E, Elsebaie MAT, Alhusseiny AM, AlMoslemany MA, Elmatboly AM, Pappalardo PA, Sakr RA, Mobadersany P, Rachid A, Saad AM, Alkashash AM, Ruhban IA, Alrefai A, Elgazar NM, Abdulkarim A, Farag AA, Etman A, Elsaeed AG, Alagha Y, Amer YA, Raslan AM, Nadim MK, Elsebaie MAT, Ayad A, Hanna LE, Gadallah A, Elkady M, Drumheller B, Jaye D, Manthey D, Gutman DA, Elfandy H, Cooper LAD (2022). NuCLS: a scalable crowdsourcing approach and dataset for nucleus classification and segmentation in breast cancer. Gigascience.

[24] Vincent BG, Szustakowski JD, Doshi P, Mason M, Guinney J, Carbone DP (2021). Pursuing better biomarkers for immunotherapy response in cancer through a crowdsourced data challenge. JCO Precis Oncol.

[25] Douzgou S, Pollalis YA, Vozikis A, Patrinos GP, Clayton-Smith J (2016). Collaborative crowdsourcing for the diagnosis of rare genetic syndromes: the DYSCERNE experience. Public Health Genomics.

[26] Brinkmann BH, Wagenaar J, Abbot D, Adkins P, Bosshard SC, Chen M, Tieng QM, He J, Muñoz-Almaraz FJ, Botella-Rocamora P, Pardo J, Zamora-Martinez F, Hills M, Wu W, Korshunova I, Cukierski W, Vite C, Patterson EE, Litt B, Worrell GA (2016). Crowdsourcing reproducible seizure forecasting in human and canine epilepsy. Brain.

[27] Marshall TF, Alfano CM, Sleight AG, Moser RP, Zucker DS, Rice EL, Silver JK, Raj VS, Fu JB, Padgett LS, Lyons KD, Radomski MV, McKenna R, Pergolotti M (2020). Consensus-building efforts to identify best tools for screening and assessment for supportive services in oncology. Disabil Rehabil.

[28] Hilton LG, Coulter ID, Ryan GW, Hays RD (2021). Comparing the recruitment of research participants with chronic low back pain using Amazon Mechanical Turk with the recruitment of patients from chiropractic clinics: a quasi-experimental study. J Manipulative Physiol Ther.

[29] Tarca AL, Pataki BÁ, Romero R, Sirota M, Guan Y, Kutum R, Gomez-Lopez N, Done B, Bhatti G, Yu T, Andreoletti G, Chaiworapongsa T, Hassan SS, Hsu CD, Aghaeepour N, Stolovitzky G, Csabai I, Costello JC, DREAM Preterm Birth Prediction Challenge Consortium (2021). Crowdsourcing assessment of maternal blood multi-omics for predicting gestational age and preterm birth. Cell Rep Med.

[30] Koepnick B, Flatten J, Husain T, Ford A, Silva DA, Bick MJ, Bauer A, Liu G, Ishida Y, Boykov A, Estep RD, Kleinfelter S, Nørgård-Solano T, Wei L, Players F, Montelione GT, DiMaio F, Popović Z, Khatib F, Cooper S, Baker D (2019). De novo protein design by citizen scientists. Nature.

[31] Sonabend AM, Zacharia BE, Cloney MB, Sonabend A, Showers C, Ebiana V, Nazarian M, Swanson KR, Baldock A, Brem H, Bruce JN, Butler W, Cahill DP, Carter B, Orringer DA, Roberts DW, Sagher O, Sanai N, Schwartz TH, Silbergeld DL, Sisti MB, Thompson RC, Waziri AE, Ghogawala Z, McKhann G (2017). Defining glioblastoma resectability through the wisdom of the crowd: a proof-of-principle study. Neurosurgery.

[32] Baldassano SN, Brinkmann BH, Ung H, Blevins T, Conrad EC, Leyde K, Cook MJ, Khambhati AN, Wagenaar JB, Worrell GA, Litt B (2017). Crowdsourcing seizure detection: algorithm development and validation on human implanted device recordings. Brain.

[33] O'Donnell S, Lewis D, Marchante Fernández M, Wäldchen M, Cleal B, Skinner T, Raile K, Tappe A, Ubben T, Willaing I, Hauck B, Wolf S, Braune K (2019). Evidence on user-led innovation in diabetes technology (the OPEN project): protocol for a mixed methods study. JMIR Res Protoc.

[34] Masselot C, Greshake Tzovaras B, Graham CLB, Finnegan G, Jeyaram R, Vitali I, Landrain T, Santolini M (2022). Implementing the co-immune open innovation program to address vaccination hesitancy and access to vaccines: retrospective study. J Particip Med.

[35] Guinney J, Wang T, Laajala TD, Winner KK, Bare JC, Neto EC, Khan SA, Peddinti G, Airola A, Pahikkala T, Mirtti T, Yu T, Bot BM, Shen L, Abdallah K, Norman T, Friend S, Stolovitzky G, Soule H, Sweeney CJ, Ryan CJ, Scher HI, Sartor O, Xie Y, Aittokallio T, Zhou FL, Costello JC, Prostate Cancer Challenge DREAM Community (2017). Prediction of overall survival for patients with metastatic castration-resistant prostate cancer: development of a prognostic model through a crowdsourced challenge with open clinical trial data. Lancet Oncol.

[36] Mason MJ, Schinke C, Eng CLP, Towfic F, Gruber F, Dervan A, White BS, Pratapa A, Guan Y, Chen H, Cui Y, Li B, Yu T, Chaibub Neto E, Mavrommatis K, Ortiz M, Lyzogubov V, Bisht K, Dai HY, Schmitz F, Flynt E, Rozelle D, Danziger SA, Ratushny A, Dalton WS, Goldschmidt H, Avet-Loiseau H, Samur M, Hayete B, Sonneveld P, Shain KH, Munshi N, Auclair D, Hose D, Morgan G, Trotter M, Bassett D, Goke J, Walker BA, Thakurta A, Guinney J, Multiple Myeloma DREAM Consortium (2020). Multiple myeloma DREAM challenge reveals epigenetic regulator PHF19 as marker of aggressive disease. Leukemia.

[37] Lewis D (2015). OpenAPS.

[38] Amgad M, Elfandy H, Hussein H, Atteya LA, Elsebaie MAT, Abo Elnasr LS, Sakr RA, Salem HSE, Ismail AF, Saad AM, Ahmed J, Elsebaie MAT, Rahman M, Ruhban IA, Elgazar NM, Alagha Y, Osman MH, Alhusseiny AM, Khalaf MM, Younes AF, Abdulkarim A, Younes DM, Gadallah AM, Elkashash AM, Fala SY, Zaki BM, Beezley J, Chittajallu DR, Manthey D, Gutman DA, Cooper LAD (2019). Structured crowdsourcing enables convolutional segmentation of histology images. Bioinformatics.

[39] Davis JR, Richard EE, Keeton K (2015). Open innovation at NASA: a new business model for advancing human health and performance innovations. Res Technol Manag.

[40] Desouza KC (2012). Challenge.gov: using competitions and awards to spur innovation. IBM Center for The Business of Government.

[41] Créquit P, Mansouri G, Benchoufi M, Vivot A, Ravaud P (2018). Mapping of crowdsourcing in health: systematic review. J Med Internet Res.

[42] Nguyen VT, Benchoufi M, Young B, Ghosn L, Ravaud P, Boutron I (2019). A scoping review provided a framework for new ways of doing research through mobilizing collective intelligence. J Clin Epidemiol.

[43] Arvaniti EN, Dima A, Stylios CD, Papadakis VG (2022). A new step-by-step model for implementing open innovation. Sustainability.

[44] HeroX.

[45] Schucht P, Roccaro-Waldmeyer DM, Murek M, Zubak I, Goldberg J, Falk S, Dahlweid FM, Raabe A (2020). Exploring novel funding strategies for innovative medical research: the HORAO crowdfunding campaign. J Med Internet Res.

[46] SurveyMonkey.

[47] Schucht P, Lee HR, Mezouar HM, Hewer E, Raabe A, Murek M, Zubak I, Goldberg J, Kovari E, Pierangelo A, Novikova T (2020). Visualization of white matter fiber tracts of brain tissue sections with wide-field imaging mueller polarimetry. IEEE Trans Med Imaging.

[48] Schucht P, Lee H, Mezouar M, Hewer E, Raabe A, Murek M, Zubak I, Goldberg J, Kovari E, Pierangelo A, Novikova T (2020). Visualization of White Matter Fiber Tracts of Brain Tissue Sections With Wide-Field Imaging Mueller Polarimetry. IEEE Trans Med Imaging.

[49] Novikova T, Pierangelo A, Schucht P, Meglinski I, Rodríguez-Núñez O, Lee HR, Ramella-Roman JC, Novikova T (2923). Mueller polarimetry of brain tissues. Polarized Light in Biomedical Imaging and Sensing: Clinical and Preclinical Applications.

[50] Rodríguez-Núñez O, Novikova T (2022). Polarimetric techniques for the structural studies and diagnosis of brain. Adv Opt Technol.

[51] McKinley R, Felger LA, Hewer E, Maragkou T, Murek M, Novikova T, Rodríguez-Núñez O, Pierangelo A, Schucht P (2022). Machine learning for white matter fibre tract visualization in the human brain via mueller matrix polarimetric data.

[52] Rodríguez-Núñez O, Schucht P, Lee HR, Mezouar MH, Hewer E, Raabe A, Murek M, Zubak I, Goldberg J, Kövari E, Pierangelo A, Novikova T (2021). Retardance map of brain white matter: a potential game changer for the intra-operative navigation during brain tumor surgery. https://tinyurl.com/mrypapzd.

[53] Rodríguez-Núñez O, Schucht P, Hewer E, Novikova T, Pierangelo A (2021). Polarimetric visualization of healthy brain fiber tracts under adverse conditions: studies. Biomed Opt Express.

[54] Carter AJ, Donner A, Lee WH, Bountra C (2017). Establishing a reliable framework for harnessing the creative power of the scientific crowd. PLoS Biol.

[55] InnoCentive.

[56] (2010). An evaluation of the open innovation pilot program between NASA and InnoCentive, Inc. INNOCENTIVE.

