# Supplementary material for: Exploring Novel Innovation Strategies to Close a Technology Gap in Neurosurgery: HORAO Crowdsourcing Campaign
Source: J Med Internet Res. 2023 Apr 28;25:e42723. doi: 10.2196/42723 (PMC10182462; doi:10.2196/42723)
Supplement: Multimedia Appendix 1 [file jmir_v25i1e42723_app1.pdf]

HORAO –
The *It Doesn't Take a Brain Surgeon* Challenge

In Greek, HORAO stands for "seeing with the mind"

Do you know a non-invasive way to visualize the human brain's fiber system? Put your mind to work and help us revolutionize brain surgery!

Guidelines Tab

Background/Overview

Overview

The HORAO Challenge aims to improve brain surgery with the help of a technology that visualizes brain fibers. The challenge seeks an idea. We welcome entrepreneurs, researchers, scientists, students, and anyone eager to contribute, to jump into this challenge and to find a solution. To register for the challenge, click the “ACCEPT CHALLENGE” button above. The Challenge Forum is your space to share thoughts and ideas with potential competitors or to create teams with similar visions.

The Problem

- Brain tumors have a devastating effects on patients and their relatives.
- For obvious reasons, brain surgery better be exact.
- Brain tumors look very similar to the normal brain, and can often not be discerned.
- Brain tissue consist of orderly fibers, whereas tumor tissue is chaotic. This difference in structure could be used to differentiate tumor from brain tissue.
- However, the fibers and their structures can not yet be seen non-invasively during surgery.

Short film presenting the HORAO problem: https://youtu.be/rVlulyBOL0c

The Human Brain

The human brain is a vital organ responsible for motion, intelligence, memory, sensation, thought, speech and emotion of a human being. It is the key to our mind and soul without which a human cannot exist. Together with the spinal cord it is part of the central nervous system and is located in the skull. The human brain makes up 2 % of the body weight and consumes 20 % of total body energy. It contains about 90 billion nerve cells, called neurons, and around the same number of supporting cells called glial cells.

The cellular body of a neuron is located in the so-called gray matter at the very surface of the brain. The extension of a neuron, called axon or nerve fiber, connects different parts of the brain and spinal cord with trillions of synapses and makes up the white matter. Thousands and millions of nerve fibers of similar function run in bundles and are

called fiber tracts. Fiber tracts always run in a predetermined way and may be many inches long.

Different brain functions are based in different areas. For example, production of speech is located in the front part of the brain while vision is located in the back part. Brain areas that house vital functions like speech, movement, memory or vision are called eloquent, while areas of no particular significance are called non-eloquent.

Tumors of the Brain and How to Treat Them

Brain tumors can occur at any age but most frequently affect children and older adults. The word tumor is used synonymous to cancer and is per definition the abnormal and uncontrolled growth of tissue that usually forms a mass. Primary brain tumors originate from brain tissue, while metastatic brain tumors begin in any given organ and then spread to the brain. Classified by their growth behavior tumors can either be benign, malignant or something in between. Benign tumors grow slowly and never spread to different tissue. Malignant tumors tend to grow very fast, destroy their surroundings and can spread elsewhere.

The most common primary brain tumor is the glioma. It arises from the glial cells, which are responsible for the support and cohesion of the neurons. Gliomas grow from within the brain and infiltrate the surrounding tissue. The malignant form of the glioma is called glioblastoma and is the most common primary malignant brain tumor in adulthood. Low grade gliomas on the other hand are tumors that grow slower, are more adapted to the brain, less destructive and therefore less distinguishable from the normal brain than a glioblastoma. However, they equally infiltrate the surrounding tissue and are thus neither benign nor malignant in terms of conventional definition.

If growing in a non-eloquent area gliomas can remain unnoticed for a very long time especially, which is often the case in low-grade of young patients. When diagnosed, the first treatment step is to remove the glioma through surgery. The surgical excision of as much of the glioma as possible is crucial to improve survival of the patient. Radiation and chemotherapy are often used to delay recurrence of the tumor.

Challenges of Brain Tumor Surgery

Most brain tumor surgery is done under general anaesthesia. Tumors close to brain areas responsible for speech or vision may require the patient to be awake in order to check the exact location of these important brain functions. The surgeons use a large, free floating binocular operation microscope that magnifies the operation site. The microscope is the surgeon’s most important ally, as it enables him to see structures more accurately, and beyond his normal vision.

Despite these abilities the microscope does not help the surgeon to distinguish between healthy brain and brain tumor. In contrast to the high organizational level of cells and fiber geometry in the brain, the appearance of brain tissue under the microscope is quite

plain. The tissue appears mostly soft (like firm jelly) and is white, while the top inch at the surface of the brain is gray, hence the terms white and gray matter as mentioned above. Especially gliomas, which are originating from the brain cells themselves, look just like brain tissue. The surgeon often struggles to see the border between the brain and the tumor even under the microscope. This problem is particularly evident in low grade gliomas which infiltrate the brain and look almost exactly like healthy brain tissue.

The most important goal of surgery is to remove as much tumor as possible without harming important brain functions. An overlooked rim of only 1 or 2 millimeters of tumor may lead to early recurrence of the tumor and reduced survival time; however, important brain areas may irreversibly be damaged if the surgeon removes 1 or 2 millimeters too much, causing devastating harm to the patient.

Three different techniques (see appendix: Neuronavigation[1], Neuromonitoring[2], 5-ALA[3]) are being used at present to aid the surgeon in finding tumor borders. But these techniques are either only estimates of the tumor borders and therefore not adequate for the high requirements of the surgery, or are only applicable in some brain tumors.

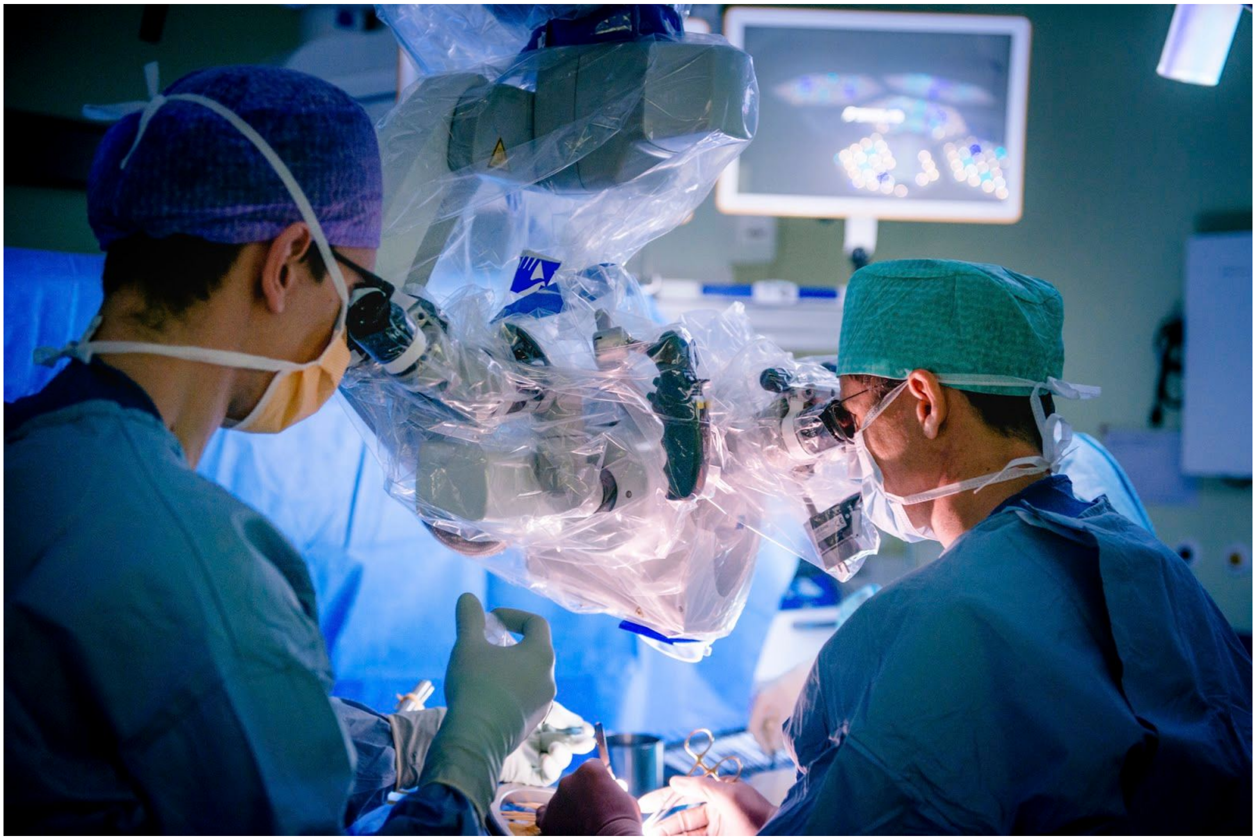

Neurosurgeons form Inselspital, Bern University Hospital operate under the microscope. (Photo: Tanja Läser for Insel Gruppe AG)

Why Does this Problem Exist?

In the quest to improve brain tumor surgery, most scientists have focused on directly visualizing tumors. Nonetheless, despite years of research on direct visualization, this strategy has failed for most types of brain tumors.

The Challenge Breakthrough

A solution to the HORAO challenge would fundamentally change todays brain surgery by providing the surgeon with real time visualization of the brains microarchitecture. It would unveil subtle differences between the human brain tissue and certain diseases yet hidden to the human eye. A challenge breakthrough would improve current surgical strategies at their base with a substantial, worldwide impact on patient safety and outcome.

We believe that in today's interconnected world of endless translational research possibilities the time is right for a solution to the HORAO challenge.

Our Commitment to a Solution

To understand the HORAO project, we have to take a step backwards: in 2017, we—a group of inspired neurosurgeons from the Inselspital, Bern University Hospital—initiated the ambitious project. We launched a crowdfunding campaign in order to make brain tumor surgery safer. It was to become one of the first successful neuroscience crowdfunding projects worldwide. Right from the start, our goal was to launch a crowdsourcing campaign as a second step, because we are convinced that there is a solution to our problem—perhaps it already exists. On March 14, 2019 up to five competitors will be invited to the HORAO Conference in Bern, Switzerland. Only then will it be decided who will win the HORAO Challenge. The conference will be attended by our jury, donors, practicing neurosurgeons, researchers, opticians, developers, and manufacturers of medical technology, which will provide a rich infrastructure and ecosystem of support for those invited to present.

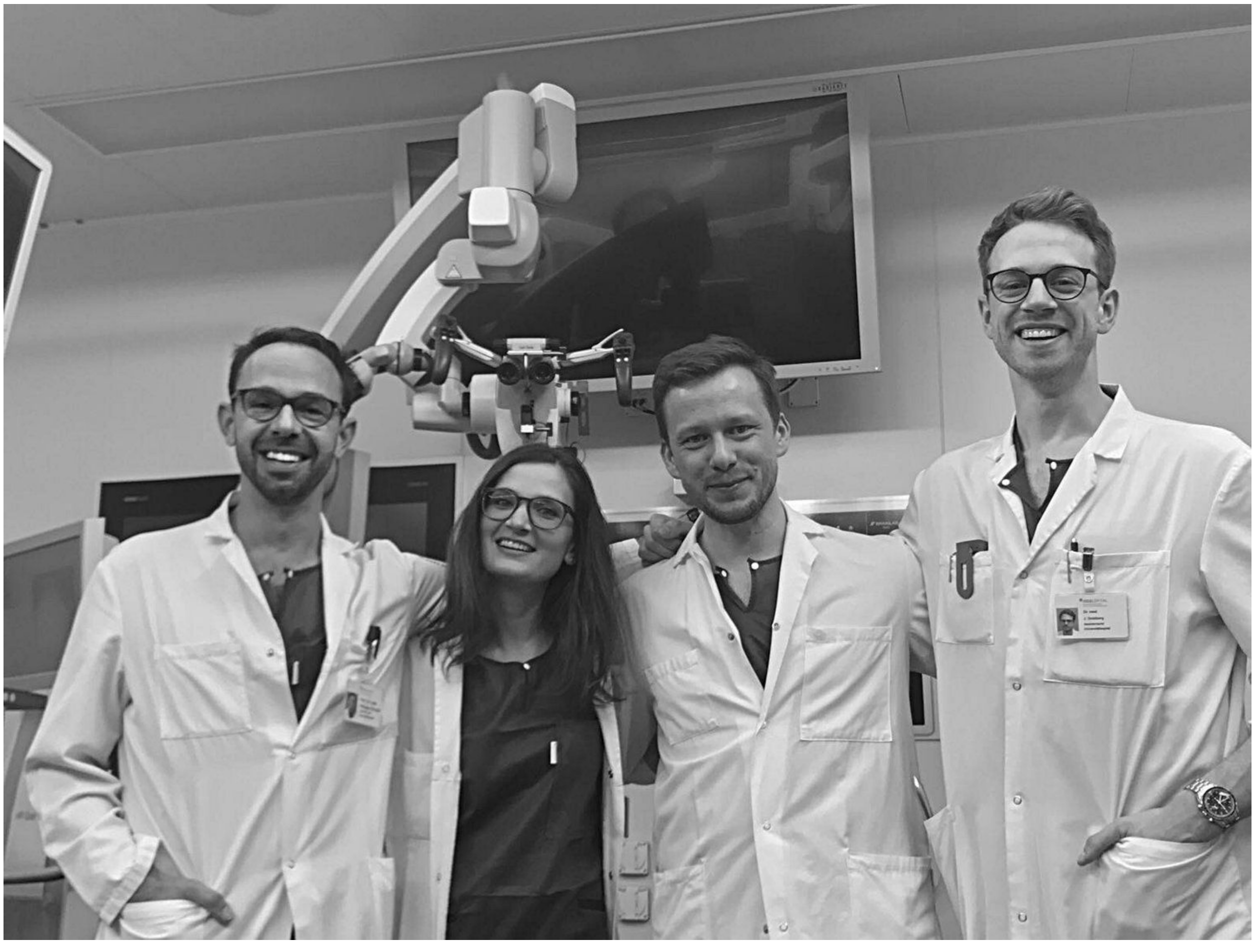

The HORAO team: Prof. Dr. med. Philippe Schucht, project manager, Dr. med. Irena Zubak, Dr. med. Michael Murek and Dr. med. Johannes Goldberg (from left to right).

Prize

1st price	$35,000
2nd price	$12,000
3rd–5th prices	$ 1,000

Up to five finalists are invited to join the final round at the HORAO Conference and convince the jury and the public of their project. The conference will be held on March 14, 2019 as a part of the Brainweek Bern in Bern, Switzerland. Finalists must present at the conference in order to compete for the prize. Travel and hotel expenses for one overnight stay will be paid for by the HORAO Challenge.

Timeline

Pre-registration begins	April 24th, 2018
Open to submissions	June 12th, 2018

Submission deadline	November 16th, 2018 @ 5pm ET
Judging	December 1st, 2018 to January 7th, 2019
Winner Announcement	January 15th, 2019
Finals (up to five participants)	March 14th, 2019

Judging Criteria

The jury assesses all submission based on the Pre-Round Judging Criteria. Up to five finalists are determined by the pre-round scores and subsequently invited to the final round. The pre-round technical judging criteria constitutes 50 % of the final score. The other 50 % is based on the presentation, content and feasibility, as judged by the jury and the public during the HORAO Conference.

Pre-Round Technical Judging Criteria

**Section**	**Description**	**Overall Weight**
Detection of cerebral tissue	The solution discerns brain from tumor tissue.	20
Detection of fiber tracts	The solution detects brain tissue in such a way that the spatial orientation of fiber tracts can be seen.	20
Real time detection	Time used for visualization must be short (minutes), in order not to disrupt the flow of surgery.	20
Size of the solution	The size of the solution must be such that it fits well into an operating theater (not larger than 2 cubic meter).	10
Non-invasiveness	The solution must not harm or remove the investigated tissue.	20
Repetitiveness	The solution must be able to be used repetitively at short interval (minutes).	10

Final Round Judging Criteria

Section	Description	Overall Weight
Score of Pre-Round	The total score obtained in the pre-round accounts for 50 % of the final score. The Jury will not communicate the Pre-Round Score.	50
Presentation, content and feasibility by jury	The jurors judge presentation, content and feasibility.	25
Presentation, content and feasibility by public vote	The public at the conference judge presentation, content and feasibility.	25

How Do I Win?

To be eligible for an award, your proposal must, at minimum:

- Satisfy the Judging Scorecard requirements
- Thoughtfully address the Submission Form questions
- Up to five top-scores are winners, and will be invited to the finals in Switzerland!

Solutions must, at a minimum:

- Help to distinguish brain tissue from tumor tissue, or:
- Identify large fiber tracts.

Rules

Participation Eligibility

The challenge is open to all adult individuals, private teams, public teams, and collegiate teams. Teams may originate from any country. Submissions must be made in English. All challenge-related communication will be in English.

No specific qualifications or expertise in the field of neurosurgery or medicine is required. Prize organizers encourage outside individuals and non-expert teams to compete and propose new solutions.

To be eligible to compete, you must comply with all the terms of the challenge as defined in the Challenge-Specific Agreement.

Registration and Submissions

Submissions must be made online (only), via upload to the HeroX.com website, on or before 5pm ET, November 16th, 2018. All uploads must be in PDF format. No late submissions will be accepted.

Intellectual Property Rights

As detailed in the Challenge-Specific Agreement, competitors will retain all intellectual property rights to their technology.

Selection of Winners

Based on the winning criteria, prizes will be awarded per the Judging Criteria section above. In the case of a tie, the winner(s) will be selected based on the highest votes from the judges.

In the event that none of the submissions meet the Judging Criteria, the sponsor will award the following consolation prizes to the competitor that score the highest:

- Consolation Prize 1: $3000
- Consolation Prize 2: $1000

Judging Panel

The determination of the winners will be made by a group of proven experts, including Prof. Dr. med. Philippe Schucht, HORAO project manager.

Additional Information

- By participating in the challenge, each competitor agrees to submit only their original idea. Any indication of "copying" amongst competitors is grounds for disqualification.
- All applications will go through a process of due diligence; any application found to be misrepresentative, plagiarized, or sharing an idea that is not their own will be automatically disqualified.

- All ineligible applicants will be automatically removed from the competition with no recourse or reimbursement.
- No purchase or payment of any kind is necessary to enter or win the competition.
- Void wherever restricted or prohibited by law.

Submission Form

**Content / Question**	**Type**	**Details**	**Character Limit**
Title*	Text (one line)	Give your submission idea a catchy title that describes the idea and gets people interested.	50
Short description*	Text (multiple lines)	Provide a brief description of your idea. Be clear and concise.	140
Image		Provide an image to represent your solution.	
Solution overview*	Text (multiple lines)	Please provide an overview of what your solution is. Be clear and concise.	1000
Explain how your solution differentiates cerebral from tumor tissue.*	Text (multiple lines)		500
Explain how your solution detects fiber tracts.*	Text (multiple lines)		500
How does your solution work in real time?*	Text (multiple lines)		500
What is the size of your solution?*	Text (multiple lines)		100
Explain how your solution works without harming or removing tissue.*	Text (multiple lines)		500
Describe how frequently your solution can be applied during one surgery.*	Text (multiple lines)		500

X-factor: Tell us what makes your solution special, what sets it apart?	Text (multiple lines)		200
Document upload / Technical report*	Upload	Please give an in-depth insight into your solution by adding technical data, sketches, plans, pictures, etc. to your proposed solution (PDF document).	5 pages max.
Link to video	Video URL		
Link to website	Link		
**Contact Information**	**Section Header**		
First name and surname*[1]	Text (one line)		
Age[1]	Dropdown (select single)	1. 15–25<br>2. 26–35<br>3. 36–45<br>4. 46–55<br>5. 56–65<br>6. 66–75<br>7. 76–85<br>8. > 85	
Gender[1]	Text (one line)		
Email*[1]	Text (one line)		
Phone number[1]	Phone number		
Geographical location*[1]	Text (one line)		
How did you hear about HORAO?	Text (multiple lines)		500
Date and time of transmission*[1]	Date and time		

[1]For administrative purposes only. Your personal data will not be forwarded to the jury.

Appendix/Additional information

1) Neuronavigation

A 3D model of the patient's head is rendered from MRI images of his skull. This model is then co-registered to the head of the patient in the operating theater. During surgery a registered probe (like a pen) can be used to virtually show a point in the patient's brain on a screen next to the operating table. This is only a virtual estimation of the true surgical situation, and subject to two important errors: Firstly the co-registration of the patients head to the MRI image is associated with a certain degree of uncertainty (up to 5mm over the course of surgery). Secondly the geometry of the brain changes as soon as the skull is opened and parts of the tumor have been resected. Mostly the brain is sinking a few millimeters into the skull and surrounding brain regions, that have been compressed by the tumor, extend after parts of the tumor have been removed. Thus the tumor and the localization of important brain areas doesn’t resemble the preoperative images any more, leading to an increasing inaccuracy as the operation goes on.

2) Neuromonitoring

The goal of neuromonitoring is to measure the function of eloquent brain areas during resection of tumors even if the patient is in general anaesthesia. In most cases the region of interest is the brain area responsible for movement of the opposite half of the body, but other areas that are responsible e.g. for visual and sensory perception can be monitored as well. With this technique the surgeon is receiving two sorts of information during the course of the operation: 1) functionality of the analyzed brain region and 2) the distance to the region.

Neuromonitoring is based upon electric principles because cells of the nervous system are sending and receiving information with electric potentials. During the operation nervous cells, for instance those responsible for movement, can be stimulated with an electric probe and the resulting activity in the muscles of the body, which are controlled by those cells, can be measured. Like this the functionality of the nervous cells is monitored. To obtain information on the distance to an eloquent brain area the electric current of the stimulating probe can be changed. One milliampere (1mA) is roughly infiltrating the brain tissue for 1mm. During the operation, for instance near the region responsible for movement, the surgeon can measure if he receives activity in the corresponding muscles after applying different strengths of electrical current. If a potential is received with 5mA we know that the distance to the nervous cells controlling that particular set of muscles is approximately 5mm.

All in all neuromonitoring is working like a compass for eloquent brain areas. Therefore it can help to decide if a resection can be continued safely near eloquent brain areas or if the resection must be stopped. However, it is not a tool to visualize the tumor itself and does not help in “in-eloquent” brain areas, where the output of the nervous cells can not be measured.

3) 5-ALA

5-ALA is a drug that is used to stain tumor cells. Patients receive this drug 4–6 hours before the operation. It is then traveling to the brain and is processed in the cells of the nervous system. Especially the malignant forms of gliomas (glioblastomas) are not able to process 5-ALA like the rest of the healthy brain, which is why a not completely processed form of 5-ALA is accumulating in glioblastoma cells. This particular form of 5-ALA can be brought to pink fluorescence after stimulation with blue light. During the operation the surgeon can switch the microscope to the blue light mode and search for pink fluorescence to distinguish healthy brain tissue from the tumor. However, 5-ALA is only staining the malignant forms of gliomas and does not work in low grade gliomas. Therefore it only helps to visualize certains types of brain tumors.
