# Supplementary material for: Exploring Novel Innovation Strategies to Close a Technology Gap in Neurosurgery: HORAO Crowdsourcing Campaign
Source: J Med Internet Res. 2023 Apr 28;25:e42723. doi: 10.2196/42723 (PMC10182462; doi:10.2196/42723)
Supplement: Multimedia Appendix 2 [file jmir_v25i1e42723_app2.pdf]

heroX

HORAO – The It Doesn’t Take a Brain Surgeon Challenge competitor agreement.

Challenge-Specific Agreement

PLEASE READ THIS CAREFULLY! You ("Innovator”) and HORAO ("Challenge Sponsor”) are entering into this Challenge-Specific Agreement ("CSA”) for this particular incentive-based competition ("Challenge”) only. In order to participate in this Challenge, Innovator must accept these terms, and therefore should take the time to understand them.

If Innovator clicks "Accept" and proceeds to register for this Challenge, this CSA will be a valid and binding agreement between Innovator and Challenge Sponsor and is in addition to the existing HeroX Terms of Use for all purposes relating to this Challenge. Innovator should print and keep a copy of this CSA. No provisions that Innovator may have agreed to that are specific to any other individual challenge will apply.

1. **Submissions.** By participating in this Challenge, Innovator may submit to Challenge Sponsor submission materials ("Submission”), as outlined in the Challenge Guidelines specific to this challenge on HeroX.com, for the Challenge to which this CSA relates. By submitting a Submission, Innovator thereby agrees to provide reasonable assistance and additional information concerning the Submission to Challenge Sponsor, if requested.
2. **Acceptance of Submission and License to Use.** Challenge Sponsor will notify Innovator if Submission has been selected for a prize ("Prize”), as outlined in the Challenge Guidelines, according to the schedule set forth in the Challenge Guidelines. Challenge Sponsor will judge all Submissions against the criteria set forth in the Challenge Guidelines and determine, in its sole discretion, which Submission best addresses the Challenge Guidelines. Challenge Sponsor has absolute and sole discretion to determine whether to accept the Innovator Submission, or any Submission, and whether to make a Prize, multiple Prizes or any Prize. The meeting of the Challenge Guidelines does not automatically mean that the Submission will be eligible for a Prize. Submissions must NOT contain or include ideas, concepts, solutions or technology in respect of which a third party owns or controls the intellectual property. Submissions and descriptions thereof may not include trademarks or trade names of corporations or entities without the permission of their owners. **By entering, Innovator represents and warrants that:**
    a. **Innovator’s entire Submission is an original work by Innovator and Innovator has not included third-party content (such as writing, text, graphics, artwork, logos, photographs, dialogue from plays, likeness of any third party, musical recordings, clips of videos, television programs or motion pictures) in or in connection with the Submission, unless (i) otherwise requested by the Challenge Sponsor and/or disclosed by Innovator in the Submission, and (ii) Innovator has either obtained the rights to use such third-party content or the content of the Submission is considered in the public domain without any limitations on use;**
    b. no person or entity other than Innovator has any right, title or interest in any part of the Submission;
    c. unless otherwise disclosed in the Submission, the use thereof by Challenge Sponsor, or the exercise by Challenge Sponsor of any of the rights granted by Innovator under this Agreement, does not and will not infringe or violate any rights of any third party or entity, including, without limitation patent, copyright, trademark, trade secret, defamation, privacy, publicity, false light, misappropriation, intentional or negligent infliction of emotional distress, confidentiality, or any contractual or other rights;
    d. Innovator has all the rights, licenses, permissions and consents necessary to submit the Submission and to grant all of the rights that Innovator has granted to Challenge Sponsor hereunder, including the right for Challenge Sponsor to use and develop derivative works of and from the Submission;
    e. all persons who were engaged by Innovator to work on the Submission or who appear in the Submission in any manner have:
        i. given Innovator their express written consent to submit the Submission for unlimited, royalty-free use, exhibition and other exploitation in any manner and in any and all media, whether now existing or hereafter discovered, throughout the world, in perpetuity;
        ii. provided written permission to include their name, image or pictures in or with the Submission (or if a minor who is not Innovator’s child, Innovator must have the permission of their parent or legal guardian) and Innovator may be asked by Challenge Sponsor to provide permission in writing;
        iii. no claims for payment of any kind, including, without limitation, for royalties or residuals;

iv. no approval or consultation rights or any rights of participation arising out of any use, exhibition or other exploitation of the Submission; and
v. not been and are not currently under any union or guild agreement that results in any ongoing obligations resulting from the use, exhibition or other exploitation of the Submission;
vi. Innovator understands, recognizes and accepts that Challenge Sponsor has access to, may create or has created materials and ideas which may be similar or identical to the Submission in concept, theme, idea, format or other respects. Innovator acknowledges and agrees that Challenge Sponsor shall have the right to use such same or similar materials, and that Innovator will not be entitled to any compensation arising from Challenge Sponsor's use of such materials. In the event that Innovator’s entry is identical or similar to the Submission of another Competitor, Challenge Sponsor reserves the right, at the sole discretion of Challenge Sponsor, to either score one Submission higher than the other subject to the Challenge Guidelines or to randomly choose a Submission from all of those submitted which respond to the Challenge Guidelines.

By entering, Innovator agrees that: (i) all Submissions become Challenge Sponsor's property and will not be returned; and (ii) Challenge Sponsor and its licensees, successors and assignees have the right to use any and all Submissions, and the names, likenesses, voices and images of all persons appearing in the Submission, for future advertising, promotion and publicity in any manner and in any medium now known or hereafter devised throughout the world in perpetuity.

All Intellectual property rights, if any, in the idea, concept, or activities demonstrated by the Submission will remain with the Innovator. The Innovator retains ownership of the Submission.

Submission may not be acknowledged and will not be received or held "in confidence." The act of submitting a Submission does not create a confidential relationship or obligation of secrecy between Innovator and any of the entities involved in this Challenge.

3. **Other Contracts, Confidentiality, and Indemnification.** Innovator agrees that Innovator will not disclose to Challenge Sponsor any information which Innovator is under an existing contractual or other legal obligation to maintain in confidence or otherwise does not have the right to sell or license. **Innovator agrees that Innovator will not discuss any confidential information of Innovator’s own, other parties, or Challenge Sponsor unless Innovator is specifically using the HeroX website to do so.** Should Innovator’s participation in the Challenge be found to breach any legal obligations Innovator may have with other third parties or in the event of a breach of the confidentiality obligations in this Section 3, Innovator agrees to defend, indemnify and hold harmless Challenge Sponsor, its and their respective officers, directors, employees, agents, licensors, and suppliers, from and against all claims, actions or demands, liabilities, and settlements, including, without limitation, reasonable legal and accounting fees, arising in connection with such unauthorized and prohibited disclosure.
4. **Changes and Cancellation.** Challenge Sponsor has the right to make updates and/or make any changes to, or to modify the scope of the Challenge Guidelines, Challenge Schedule, and Winning Criteria at any time during the Challenge. Innovators are responsible for regularly reviewing the Challenge site to ensure they are meeting all rules and requirements of the Challenge. Challenge Sponsor has the right to cancel the Challenge at any time, without warning or explanation, and to subsequently remove the Prize completely.
5. **General Conditions.** Challenge Sponsor has the right to verify each Innovator's eligibility and compliance with this CSA. Challenge Sponsor has the right to enforce the terms and conditions hereof directly against Innovator. Participation is conditioned on providing the data required in the online registration form. Innovators should direct any request to access, update, or correct information to Challenge Sponsor. Challenge Sponsor is not responsible for human error, theft, destruction, or damage to Submissions, or other factors beyond its reasonable control.
    a. Innovators should not register with multiple e-mail and/or street addresses. In the event of a dispute as to any Submission, the authorized account holder of the email address used to enter will be deemed to be the person who submitted the Submission. The authorized "account holder" is the natural person assigned an email address by an Internet access provider, online service provider or other organization responsible for assigning email addresses for the domain associated with the submitted address;
    b. Innovator is responsible for any related costs to complete activities related to solving the challenge;
    c. Innovator is responsible for obeying any local, national, and international laws in undertaking any activities related to solving the challenge;
    d. Innovator is responsible for obtaining any insurance related to activities performed to solve the challenge;
    e. Innovator is responsible for truthful representation of all activities undertaken to solve the challenge; Challenge Sponsor reserves the right, at any time, to verify such activities;
    f. Innovator is responsible for maintaining accurate records and truthfulness of metrics, or impact related to activities undertaken to solve the challenge; Challenge Sponsor is not responsible for calculating results, but reserves the right, at any time, to verify such information;

g. Innovator is responsible for completing registration and submitting Submissions by the dates outlined in the Challenge Guidelines; Challenge Sponsor is not required to accept late registrations or late Submissions;
h. Challenge Sponsor is not liable for any activities undertaken to solve the challenge;
i. Challenge Sponsor has the right to disqualify an individual from the challenge at any time.

6. **Representations and Warranties.** Innovator represents and warrants that:
   a. All information provided by Innovator regarding Innovator and, if applicable, its business ("Competitor Information") is true, accurate, current, and complete information and Innovator will maintain and update the Competitor Information to keep it true, accurate, current and complete.
   b. If Innovator is an individual representing a business or other entity, Innovator is authorized to enter into this Agreement on behalf of that business or entity.
   c. Unless otherwise disclosed in the Submission, Innovator is the owner of the Submission and the Submission does not infringe or violate any patent, copyright, trade secret, trademark or other third-party intellectual property right.
   d. Innovator has the right to grant the license in the Submission as required by Section 2 of this CSA.
7. **Conflict.** In the case of any conflict between the terms of this CSA and the Terms of Use, this CSA controls.
8. **Governing Law and Venue**. This agreement will be governed and interpreted by and under the laws of Switzerland. Both parties agree that any cause of action arising under this Agreement shall be brought exclusively settled by the Court of Berne, Switzerland.
