# Supplementary material for: Exploring Novel Innovation Strategies to Close a Technology Gap in Neurosurgery: HORAO Crowdsourcing Campaign
Source: J Med Internet Res. 2023 Apr 28;25:e42723. doi: 10.2196/42723 (PMC10182462; doi:10.2196/42723)
Supplement: Multimedia Appendix 3 [file jmir_v25i1e42723_app3.pdf]

Submission Form Preview

Section 1: **Introduction**

Title *

Give your submission a catchy title that describes the idea and gets people interested.

0/50

Short description

Provide a brief description of your idea. Be clear and concise.

0/140

Image

An Image boosts your message by illustrating your solution. For best results, ensure your image contains the following items: an actor(ess) (person), artifact (tool they're holding), action (what they're doing), and atmosphere (setting where they are). Ensure your image is at least 650 pixels wide by 366 pixels tall for clarity.

Supported File Types: **PNG, JPG**

Upload Image

How did you hear about this challenge?

0/100

Section 2: **Content / Question**

Solution overview *

Please provide an overview of what your solution is. Be clear and concise.

0/1000

Explain how your solution differentiates cerebral from tumor tissue. *

0/500

Explain how your solution detects fiber tracts. *

0/500

How does your solution work in real time? *

0/500

What is the size of your solution? *

0/100

Explain how your solution works without harming or removing tissue. *

0/500

Describe how frequently your solution can be applied during one surgery. *

0/500

X-factor: Tell us what makes your solution special, what sets it apart?

0/200

Document upload / Technical report *

please give an in-depth insight into your solution by adding technical data, sketches, plans, pictures, etc. to your proposed solution (pdf document, 5 pages max)

Supported File Types: **PDF**

Choose File

Link to video

Link to website

Section 3: **Contact information**

For administrative purposes only. Your personal data will not be forwarded to the jury.

First name and surname *

For administrative purposes only. Your personal data will not be forwarded to the jury.

0/3000

Age

For administrative purposes only. Your personal data will not be forwarded to the jury.

Gender

For administrative purposes only. Your personal data will not be forwarded to the jury.

0/3000

Email *

For administrative purposes only. Your personal data will not be forwarded to the jury.

0/3000

Phone number

For administrative purposes only. Your personal data will not be forwarded to the jury.

0/3000

Geographical location *

For administrative purposes only. Your personal data will not be forwarded to the jury.

Enter location

How did you hear about HORAO?

For administrative purposes only. Your personal data will not be forwarded to the jury.

0/500

Date and time of transmission *

© Copyright HeroX 2021

Was this page helpful?
Yes No
